# Supplementary material for: Plasma B Vitamers: Population Epidemiology and Parent-Child Concordance in Children and Adults
Source: Nutrients. 2021 Mar 2;13(3):821. doi: 10.3390/nu13030821 (PMC8001009; doi:10.3390/nu13030821)
Supplement: Supplementary file 1 [file nutrients-13-00821-s001.pdf]

Supplementary material

Supplementary Table S1: List of materials	
**Material**	**Purchased from**
LC-grade Acetonitrile	LiChrosolv (Germany)
LC-grade acetic acid	EMSURE (Germany)
Heptafluorobutyric acid (HFBA)	Sigma Aldrich
Bovine serum albumin (BSA)	Sigma Aldrich
Phosphate buffer saline (PBS)	Sigma Aldrich
Ascorbic acid	Scharlau Chemie (Barcelona).
Impact protein precipitation 96-well plate	Phenomenex®
96-well collection plate (square 2ml/well)	Phenomenex®
Eppendorf tips (50, 300 and 1000µL)	Eppendorf

Supplementary Table S2: Internal standard solution composition			
Internal standard	Solvent	Target Concentration (µM)	Volume of internal standard (µL)
**Nicotinamide-2, 4, 5, 6-d4**	HcL 0.1 mol/L	4000	12.5
**Pyridoxine-d2 HCl (5-Hydroxymethyl-d2)**	HcL 0.1 mol/L	2500	12.5
**Biotin d4**	EtOH/water 1:1	2000	12.5
**Thiamine hydrochloride 13C3**	HcL 0.1 mol/L	1500	33.3
**Riboflavin 13C4**	H2O, Ac.ac 0.12%	600	41.7
**Pyridoxamine d3**	HcL 0.1 mol/L	2000	12.5
**Pantothenic acid 13C6 15N2**	H2O	1000	100
**Folic acid 13C5**	5%NaOH 0.1mol/L, 20%EtOH in water	500	50
**Nicotinic acid d4**	HcL 0.1 mol/L	2000	12.5
**Nicotinuric acid d4**	HcL 0.1 mol/L	2000	12.5
**Trimethylamine N-oxide 13C3**	H2O	2000	250

Supplementary Table S3: Calibration curve standard solution composition			
**Standard**	**Solvent**	**Target Con(μM)**	**Volume of most concentrate standard *(μL)**
**Pyridoxal 5'-phosphate monohydrate**	HcL 0.1 mol/L	2000	75
**Nicotinic Acid**	HcL 0.1 mol/L	4500	33.3
**4-Pyridoxic Acid**	HcL 0.1 mol/L	2000	75
**Nicotinamide**	HcL 0.1 mol/L	10000	60
**FMN Riboflavin 5'-Phosphate**	H2O, Ac.ac 0.12%	1000	60
**Pyridoxal**	HcL 0.1 mol/L	3000	30
**Pyridoxine**	HcL 0.1 mol/L	2500	24
**Pyridoxamine**	HcL 0.1 mol/L	2300	26.1
**Riboflavin**	H2O, Ac.ac 0.12%	400	75 (Dilute stock 1:2 first)
**Thiamine**	HcL 0.1 mol/L	1700	35.3
**Biotin**	EtOH/water 1:1	2000	30 (Dilute stock 1 :10 first)
**Pantothenic Acid**	H2O	4500	133.3
**Nicotinuric Acid**	HcL 0.1 mol/L	2800	53.6
**Folic acid**	5%NaOH 0.1mol/L, 20%EtOH in H20	1000	150
**Trimethylamine N-oxide dihydrate**	H2O	10000	300
**The most concentrated standard undergoes a serial dilution to create a calibration curve with a large dynamic range of concentrations*			

Supplementary Table S4: Stock solution water dilutions									
**Standard**	**S’1**	**S’2**	**S’3**	**S’4**	**S’5**	**S’6**	**S’7**	**S’8**	**S’9**
**Volume of H2O µL**	160	180	180	190	180	160	120	60	no prep
**Volume of Stock µL**	40 of S3	20 of S5	20 of S6	10 of S9	20 of S9	40 of S9	80 of S9	140 of S9	

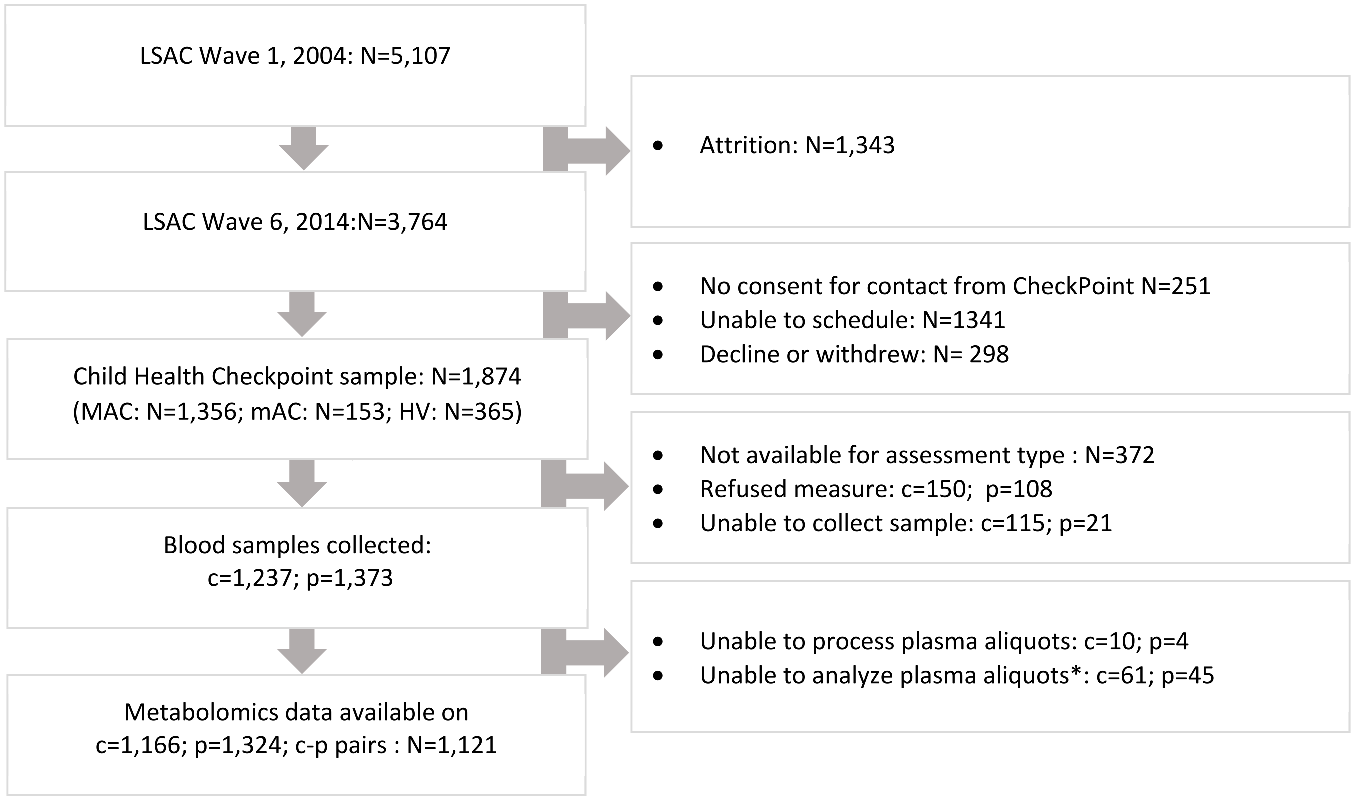

Supplementary Figure S1: Participant flow chart.

*Unable to analyse due to insufficient volume or poor-quality sample. HV: home visit; LSAC: Longitudinal Study of Australian Children; MAC: main assessment centre; mAC: mini assessment centre; N: number of families; p: number of attending adults; c: number of attending children.

Supplementary Table S5: Linear model results of age-specific differences in B vitamer concentrations in the adult subgroup			
**Vitamer***	**Estimate**	**Adjusted R^2 of linear model**	**p value**
**Thiamine (B1)**	0.01	0.002	0.07
**Riboflavin (B2)**	0.01	0.004	0.01
**FMN (B2)**	0.001	-0.0005	0.58
**Nicotinamide (B3)**	-0.002	-0.0003	0.42
**Pantothenic acid (B5)**	0.02	0.025	<0.0001
**4-Pyridoxic acid (B6)**	0.01	0.003	0.02

Log transformed variables
